# Chronic Valproic Acid Administration Increases Plasma, Liver, and Brain Ammonia Concentration and Suppresses Glutamine Synthetase Activity

**DOI:** 10.3390/brainsci10100759

**Published:** 2020-10-21

**Authors:** Abdelnaser A. Badawy, Rasha Elghaba, Mohamed Soliman, Abdelaziz M. Hussein, Sana A. AlSadrah, Amira Awadalla, Osama A. Abulseoud

**Affiliations:** 1 Department of Biochemistry, Faculty of Medicine, Northern Border University, Arar 73213, Saudi Arabia; abdelnaserbadawy_bio@yahoo.com; 2 Department of Biochemistry, Faculty of Medicine, Mansoura University, Mansoura 35516, Egypt; 3 Department of Medical Physiology, Faculty of Medicine, Mansoura University, Mansoura 35516, Egypt; r.soliman80@yahoo.com; 4 Department of Microbiology, Faculty of Medicine, Northern Border University, Arar 73213, Saudi Arabia; msolimanlab2012@gmail.com; 5 Department of Preventive Medicine, Governmental Hospital Khobar, Health Centers in Khobar, Ministry of Health, Khobar 34446, Saudi Arabia; sana12345.uk@yahoo.com; 6 Center of Excellence and Cancer Genome, Mansoura Urology and Nephrology Center, Mansoura 35516, Egypt; a.lahlouba@hotmail.com; 7 Neuroimaging Research Branch, IRP, National Institute on Drug Abuse, National Institutes of Health, Biomedical Research Center, Baltimore, MD 21224, USA

**Keywords:** valproic acid, ammonia, hyperammonemia, glutamine synthetase, striatum, prefrontal cortex, cerebellum, mechanism

## Abstract

Asymptomatic valproic acid (VPA)-induced hyperammonemia in the absence of liver impairment is fairly common. However, the underlying mechanisms through which VPA causes elevation in plasma ammonia (NH_4_) remains under investigation. Male Sprague Dawley rats (*n* = 72) were randomly allocated to receive VPA 400 mg/kg, 200 mg/kg, or vehicle IP daily for either 8, 14, or 28 consecutive days. The behavioral effects of VPA were assessed. Plasma, liver, and prefrontal cortex (PFC), striatum (Str), and cerebellum (Cere) were collected 1 h post last injection and assayed for NH_4_ concentration and glutamine synthetase (GS) enzyme activity. Chronic VPA treatment caused attenuation of measured behavioral reflexes (*p* < 0.0001) and increase in plasma NH_4_ concentration (*p* < 0.0001). The liver and brain also showed significant increase in tissue NH_4_ concentrations (*p* < 0.0001 each) associated with significant reduction in GS activity (*p* < 0.0001 and *p* = 0.0003, respectively). Higher tissue NH_4_ concentrations correlated with reduced GS activity in the liver (*r* = −0.447, *p* = 0.0007) but not in the brain (*r* = −0.058, *p* = 0.4). Within the brain, even though NH_4_ concentrations increased in the PFC (*p* = 0.001), Str (*p* < 0.0001), and Cere (*p* = 0.01), GS activity was reduced only in the PFC (*p* < 0.001) and not in Str (*p* = 0.2) or Cere (*p* = 0.1). These results suggest that VPA-induced elevation in plasma NH_4_ concentration could be related, at least in part, to the suppression of GS activity in liver and brain tissues. However, even though GS is the primary mechanism in brain NH_4_ clearance, the suppression of brain GS does not seem to be the main factor in explaining the elevation in brain NH_4_ concentration. Further research is urgently needed to investigate brain NH_4_ dynamics under chronic VPA treatment and whether VPA clinical efficacy in treating seizure disorders and bipolar mania is impacted by its effect on GS activity or other NH_4_ metabolizing enzymes.

## 1. Introduction

Valproic acid (VPA) is one of the most widely used medications for different types of seizures [1,2] bipolar mania [3], and migraine headache prophylaxis [4]. The clinical use of VPA is associated with a wide range of adverse events from mild nausea and vomiting to hepatotoxicity and pancreatitis [5]. However, VPA-induced elevation in plasma ammonia (NH_4_) concentration is one of the most intriguing side effects of VPA in patients with psychiatric disorders or epilepsy. A review of 24 studies reported that the prevalence of VPA-associated hyperammonemia ranged between 70% and 100% in prospective studies and between 16% and 100% in cross-sectional studies [6]. One retrospective chart review for 347 patients admitted to a psychiatric unit reported the incidence of VPA-hyperammonemia is about 36%, with 43.2% of those patients with VPA-induced hyperammonemia presenting with symptoms [7]. This incidence is very close to the 27.8% incidence reported in 158 patients with epilepsy [8]. However, other studies reported higher rates of 72.5% (27/40) in elderly psychiatric patients [9] and in patients with seizure disorder 52% (29/55) [10]. VPA-induced hyperammonemia is also reported during VPA loading dose (20 or 30 mg/kg) at 6 or 10 mg/kg/min, one-hour post-VPA infusion. Plasma NH_4_ doubled reaching 92.5 ± 38.2 µmol/L at 60 min and returned to baseline concentration at 24 h in 66% of cases [11]. Hyperammonemia has also been reported in a Chinese cohort of 21 patients with seizure disorder undergoing VPA treatment. The mean NH_4_ level was 138 ± 68 µmol/L [12]. In the vast majority of these studies, over 50% of patients remains asymptomatic, and, in those who present with encephalopathy, the level of NH_4_ does not seem to correlate with the severity of symptoms [6,13,14]. Equally important, liver functions were all within normal range, which raises the question about the mechanism of hyperammonemia [15].

Under normal physiological conditions, NH_4_ is generated in the gut through amino acid catabolism in the intestinal mucosal cells and by the bacterial microflora in the colon [16]. This NH_4_ is handled by liver hepatocytes through two different systems: the high-capacity, low-affinity urea cycle and the high-affinity, but low-capacity glutamine synthetase (GS) system. These two systems provide effective means of metabolizing NH_4_ delivered to the liver and ensuring low levels of NH_4_ reaching systemic circulation [17].

The kidney is another source of NH_4_ generation through the hydrolysis of glutamine in the proximal renal tubules by glutaminase enzyme. One study on 20 patients showed that the administration of 1500 mg VPA provoked in the kidney an increased glutamine uptake correlated with an increased NH_4_ release, as shown by the changes of the renal arterial–venous concentration differences of glutamine and NH_4_ [18]. This increase in NH_4_ is due to the activation of the glutaminase enzyme as shown by incubating VPA (0.01–10 mM) with human kidney cortex tubule slices for 60 min [19].

These data suggest that the kidneys contribute to VPA-induced hyperammonemia. However, the elevation in plasma NH_4_ could also be due to reduced NH_4_ clearance in other organs such as the liver and the brain. Since the human kidney cortex is devoid of GS activity [20] and urea cycle enzyme activities in the liver do not show changes reflecting inability of the liver to detoxify ammonia during VPA-induced hyperammonemia [21], the removal of excessive NH_4_ through incorporation into glutamine by glutamine synthetase (GS) enzyme in the liver and brain should be examined.

In the brain, NH_4_ is generated during glutamatergic and GABAergic neurotransmission by the phosphate-activated glutaminase enzyme (PAG), which generates NH_4_ and glutamate from glutamine [22]. Another portion of brain NH_4_ comes through diffusion from the plasma across the blood–brain barrier. Normally, the ratio of brain to blood NH_4_ ranges between 1.5:1 and 3.0:1 [23]. Brain NH_4_ is maintained at low concentrations through efficient clearance by the GS enzyme [23,24]. In addition, high brain NH_4_ could diffuse back into the blood [25] and cause elevation in plasma ammonia if the limited capacity of the GS enzyme is exceeded.

We have recently shown that transient elevation in plasma NH_4_ could originate from the brain after a single injection of tetrahydro-cannabinoid (THC) due to the suppression of striatal GS enzyme activity [26]. Similarly, a VPA 1.2 mM application to astrocyte cell culture was associated with 30% reduction in GS activity [27]. Furthermore, GS gene polymorphism (*GLUL* rs10797771) had significant associations with plasma NH_4_ level elevation during VPA treatment in a cohort of 202 Japanese pediatric patients with epilepsy [28]. The effect of VPA on GS activity depends on several factors. For example, hippocampal GS activity increased by 43% in male rats prenatally exposed to VPA when examined at post-natal day 15, but VPA caused significant reduction (27%) in GS activity at post-natal day 120 [29]. As such, the complex effects of VPA on brain GS activity and its potential contribution to the elevation in plasma NH_4_ is not entirely clear. In this study, we hypothesized that chronic VPA administration will induce elevation in plasma, brain, and liver NH_4_ concentration and concomitant reduction in GS activity.

## 2. Material and Methods

### 2.1. Animals

All experimental procedures were approved by and conducted according to the guidelines of the institutional animal care committee of Mansoura College of Medicine (# r/17.01.102). Eighty male Sprague Dawley rats, aged 12–16 weeks and weighing 200–250 g at the beginning of the study were used. Rats were individually housed in separate cages with a free supply of food (ad libitum) and tap water under a 12:12 light:dark cycle, with lights turned on at 6 a.m. Behavioral assessments were done during the light phase.

### 2.2. Study Design

Three main experimental groups (*n* = 24 each) were used; vehicle control, VPA 200 mg/kg, and VPA 400 mg/ kg. Each main group had three subgroups (*n* = 8 each) depending on the duration of VPA administration at 8, 14, and 28 days. Two groups of rats (*n* = 4 each) were used to measure VPA plasma level at 20 min (peak) and at 12 h (trough) post 400 mg/kg VPA administration.

Valproic acid sodium salt was purchased from Sigma-Aldrich (St. Louis, MO, USA, catalog # P4543-100G) and dissolved in saline for intraperitoneal (IP) injection (0.5 mL). Vehicle control group received IP injection of 0.5 mL of saline.

### 2.3. Behavioral Testing

The effect of chronic VPA administration on the level of rat alertness was assessed using the Irwin scoring system [30], which was originally used to assess animal models of hyperammonemia. Irwin’s scale includes the following items (a) Corneal reflex: 0 = none, 2 = sluggish closure, 4 = active single eye-blink, 6 = double eye-blink, and 8 = triple eye-blink; (b) Pinna reflex: 0 = none, 2 = moderate retraction, 3 = slight brisk flick, 4 = active retraction, 5 = moderate brisk flick, 6 = very brisk flick, and 8 = hyperactive repetitive flick barely touch with body withdrawal response; (c) Positional passivity: 0 = no struggle, 2 = held by neck, 4 = held supinely, 6 = held by foreleg, and 8 = held by hindlimbs; and (d) Pain (paw pressure) reflex: 0 = none, 2 = slight withdrawal, 4 = moderate rapid withdrawal, not brisk, 6 = brisk, rapid withdrawal, and 8 = very brisk withdrawal repetitive extension and flexion. Lower total or individual scores indicate more behavioral impairment. Behavioral assessment was done within 5 min from the last VPA administration.

### 2.4. Animal Euthanasia and Blood Sample Collections and Harvesting of Brain and Liver Tissues

Rats were anesthetized at 60 min post last VPA administration by halothane inhalation, and then euthanized by cervical decapitation. Trunk blood samples were collected in pre-cooled (4 °C) heparinized tubes and centrifuged at 10,000× *g* for 3 min. Plasma was used immediately for colorimetric ammonia assay as detailed before [26]. Immediately after the decapitation, the brain (prefrontal cortex (PFC), striatum (Str), and cerebellum (Cere)) and the liver tissues were dissected on ice water (4 °C) under light microscopy and transferred on dry ice to a liquid nitrogen till assay.

### 2.5. VPA Plasma Concentration

VPA assay was performed through chemiluminescent microparticle immunoassay following the manufacturer’s protocol (Abbott Laboratories, Lake Bluff, IL, USA).

### 2.6. Ammonia Determination in Plasma, Liver, and Brain Tissues

We followed the same methods detailed in our previous report. Briefly, rat plasma (25 µL) was deproteinized with an equal volume of 8% perchloric acid and centrifuged at 4000× *g* (4 °C) for 5 min. Specimens were neutralized with 2 M potassium bicarbonate and re-centrifuged at 4000× *g* (4 °C) for 10 min prior to analysis. Following the last collection, specimens were analyzed as mentioned above.

Brain and liver tissues were homogenized in 20 times *w*/*v* of ice-cold ammonia kit buffer (BioVision^®^ Milpitas, CA, USA) and centrifuged at 4000× *g* (4 °C) for 5 min. The ammonia concentration was determined using a commercial ammonia colorimetric assay kit (Biovision^®^) [26].

### 2.7. Glutamine Synthetase (GS) Activity in Liver and Brain Tissues

By using a mortar and pestle, about 20–50 mg of brain and liver tissues were homogenized in 1–2 mL cold buffer (50 mm potassium phosphate, pH 7.5, 1 mm EDTA) and centrifuged for 15 min at 4000× *g* rpm (4 °C). The activity of GS (U/g tissues) was measured in the supernatant of brain and liver homogenates using commercially available kits (MyBio-Source, San Diego, CA, USA MBS8243181), according to the manufacturer’s instructions.

### 2.8. Statistical Analysis

All data were presented as mean ± SEM (standard error of the mean). Separate two-factor analyses of variance (ANOVAs) with VPA treatment (vehicle vs. 200 mg/kg/d vs. 400 mg/kg/d) and treatment duration (8 vs. 14 vs. 28 days) were used to examine the effect of VPA on behavioral and molecular variables. Tukey’s multiple comparisons tests were used to examine differences between individual groups when ANOVA showed significant effects. Pearson correlations were utilized to examine the relationships between plasma NH_4_ and behavioral scores and between tissue NH_4_ concentrations and GS activities. Analysis was performed using GraphPad Prism V8 software. Results are considered significant at *p* < 0.05.

## 3. Results

### 3.1. Behavioral Effects of Chronic VPA Treatment

**Figure 1:**
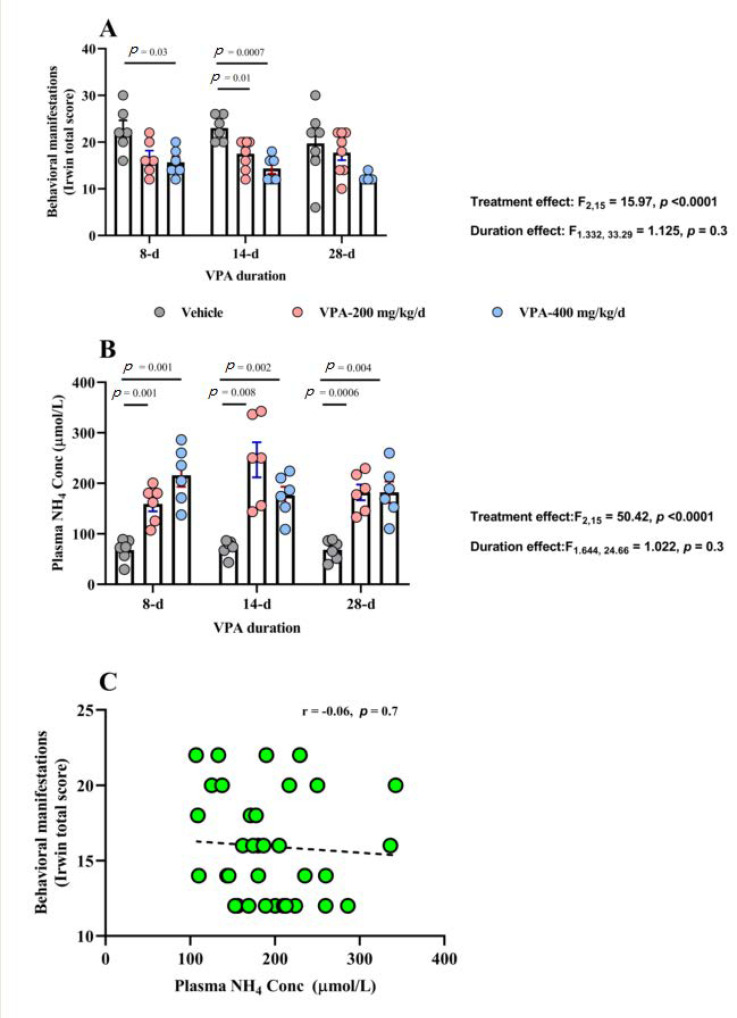
The effect of VPA on behavioral manifestations and plasma ammonia (NH_4_) concentration. (**A**) VPA treatment shows significant effect on Irwin’s total score: F_2,50_ = 15.97, *p* < 0.0001 by two-way ANOVA (*n* = 6/group). Significant differences were observed at 8 d of treatment between vehicle and VPA 400 mg/kg/d (mean difference, 95% CI, *p* value: 7.00, 0.4291 to 13.57, *p* = 0.037) and at 14 days of treatment between vehicle and VPA 200 mg/kg/d (mean difference, 95% CI, *p* value: 5.50, 1.250 to 9.750, *p* = 0.012) and at 28 d of treatment between vehicle and VPA 400 mg/kg/d (mean difference, 95% CI, *p* value: 5.417, 0.6275 to 10.21, *p* = 0.029) by Tukey’s multiple comparisons test. (**B**) VPA treatment has significant effect on plasma NH_4_ concentration: F_2,15_ = 50.42, *p* < 0.0001 by two-way ANOVA (*n* = 6/group). Significant differences were observed at 8 d of treatment between vehicle and VPA 200 mg/kg/d (mean difference, 95% CI, *p* value: −91.34, −140.2 to −42.50, *p* = 0.001) and between vehicle and VPA 400 mg/kg/d (mean difference, 95% CI, *p* value: −147.9, −221.1 to −74.66, *p* = 0.001), and at 14 d of treatment between vehicle and VPA 200 mg/kg/d (mean difference, 95% CI, *p* value: −173.9, −286.1 to −61.71, *p* = 0.008) and between vehicle and VPA 400 mg/kg/d (mean difference, 95% CI, *p* value: −103.6, −158.3 to −48.97, *p* = 0.002), and at 28 d of treatment between vehicle and VPA 200 mg/kg/d (mean difference, 95% CI, *p* value: −113.6, −164.4 to −62.77, *p* = 0.0006) and between vehicle and VPA 400 mg/kg/d (mean difference, 95% CI, *p* value: −113.8, −181.6 to −46.05, *p* = 0.004) by Tukey’s multiple comparisons test. (**C**) Significant negative correlation between total Iriwn’s scale scores and plasma ammonia (NH_4_) concentration: *r* = −0.4498, *p* = 0.0006.

VPA treatment was associated with significant attenuation in Irwin’s total score, (*p* < 0.0001). No effect for duration (*p* = 0.3) and no treatment × duration interaction (*p* = 0.6) was found. (Table 1 and Figure 1A). Tukey’s multiple comparisons test showed significant differences between vehicle and VPA 200 mg/kg/d at 14 d (*p* = 0.012) and between vehicle vs. VPA 400 mg/kg/d at 8 d (*p* = 0.037) and at 14 d (*p* = 0.0007) but not at 28 d (*p* = 0.09).

### 3.2. VPA Plasma Concentration

Peak and trough VPA concentrations were measured in a subgroup of animals (*n* = 4) at (peak at 20 min post 400 mg/kg dose = 250 ± 28.87 µg/mL and trough at 12 h post dose: 6.21 ± 1.63 µg/mL.

### 3.3. Effect of Chronic VPA Treatment on Plasma NH_4_ Concentration

A robust VPA treatment effect was evident on plasma NH_4_ concentration (*p* < 0.0001), with significant interaction between VPA treatment and duration (*p* = 0.03). However, we did not see effect for duration alone (*p* = 0.3, Figure 1B and Table 2). Tukey’s multiple comparisons test showed both VPA dose groups separated from vehicle group at all three time points: vehicle vs. VPA 200 mg /kg/d at 8 d (*p* = 0.0016), at 14 d (*p* = 0.008), and at 28 d (*p* = 0.0006) and similarly, vehicle vs. VPA 400 mg/kg/d at 8 d (*p* = 0.0017), at 14 d (*p* = 0.002), and at 28 d (*p* = 0.004).

In addition, we did not observe significant correlation between plasma NH_4_ concentration and Irwin’s behavioral scores within hyperammonemia animals only (r = −0.06, *p* = 0.7, Figure 1C).

To explore the source for the elevation in plasma NH_4_, we next examined liver and brain NH_4_ concentrations and GS activities.

### 3.4. Effect of Chronic VPA Treatment on Liver NH_4_ Concentration and GS Activity

**Figure 2:**
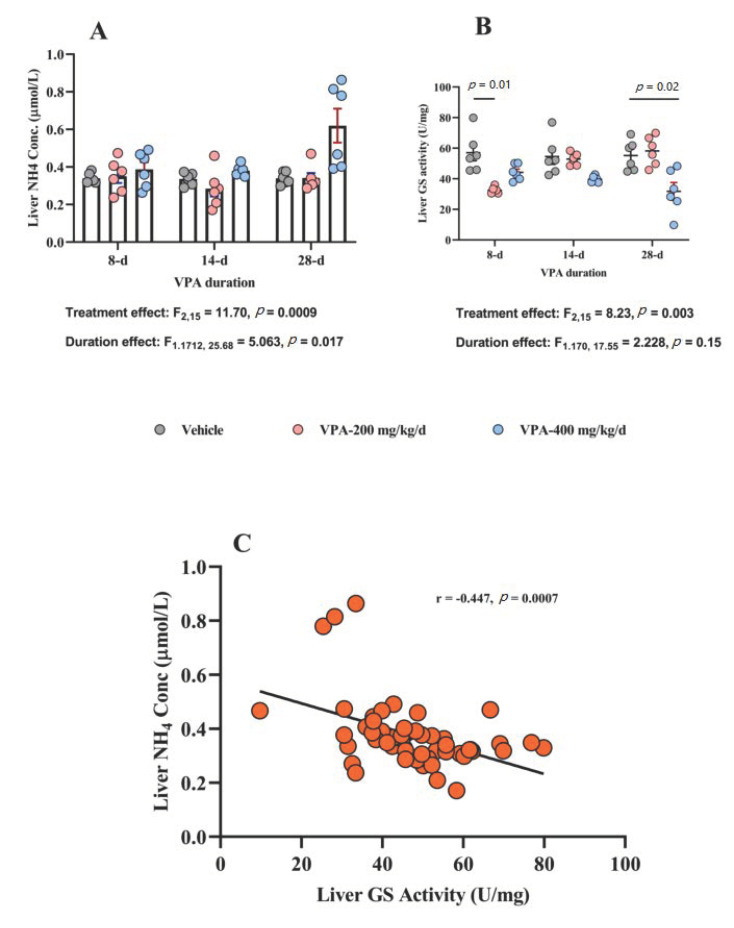
The effect of VPA on liver ammonia (NH_4_) concentration and glutamine synthetase (GS) enzyme activity. (**A**) VPA treatment (F_2,15_ = 11.70, *p* = 0.0009) and duration (F_1.712,25.68_ = 5.063, *p* = 0.017) have significant effects on liver NH_4_ by two-way ANOVA (*n* = 6/group). However, no significant differences were observed at any of the three time points between vehicle and VPA 200 or 400 mg/kg/d. Only a trend toward significance (*p* = 0.07) was found at 14 days for the 200 mg/kg/d group (vs. vehicle) and at 28 days for both 200 mg/kg/d group (vs. vehicle, *p* = 0.059) and also the 400 mg/kg/d group (vs. vehicle, *p* = 0.059) by Tukey’s multiple comparisons test. (**B**) Only VPA treatment had significant effect on liver GS activity: F_2,15_ = 8.23, *p* = 0.003 by two-way ANOVA (*n* = 6/group). Significant differences were observed at 8 d of treatment between vehicle and VPA 200 mg/kg/d (mean difference, 95% CI, *p* value: 24.56, 7.610 to 41.51, *p* = 0.011) and at 14 d of treatment between vehicle and VPA 400 mg/kg/d (mean difference, 95% CI, *p* value: 13.26, 8.062 to 18.45, *p* = 0.0003) and at 28 d of treatment between vehicle and VPA 400 mg/kg/d (mean difference, 95% CI, *p* value: 23.46, 3.780 to 43.14, *p* = 0.02) by Tukey’s multiple comparisons test. (**C**) Significant negative correlation was found between liver NH_4_ concentration and liver GS activity: *r* = −0.447, *p* = 0.0007.

VPA treatment was associated with overall significant treatment (*p* = 0.009) and duration (*p* = 0.01) effects on liver NH_4_ concentration and treatment × duration interaction (*p* = 0.01). However, Tukey’s multiple comparisons test did not show significant differences between individual groups at the three tested time points (Table 2 and Figure 2A). On the other hand, GS activity was reduced by VPA treatment (*p* = 0.003) independent of treatment duration (*p* = 0.1) with a significant treatment × duration interaction (*p* < 0.001). Here, we observed that the lower VPA dose (200 mg/kg/d) was associated with less GS activity compared to vehicle at the short (8 d) treatment duration (*p* = 0.01) and the higher VPA dose (400 mg/kg/d) at the long (28 d) treatment duration (*p* = 0.02) by Tukey’s multiple comparisons test (Figure 2B).

Next, we examined the correlation between the increase in liver NH_4_ concentration and the reduction in GS activity and found a significant negative correlation (*r* = −0.447, *p* = 0.0007, Figure 2C).

Here, we thought that VPA-induced hyperammonemia could have hepatic origins through inhibition of hepatic GS activity. However, this does not fully explain the associated behavioral effects we observed earlier, so we examined whether brain NH_4_ concentration is also elevated through a similar mechanism (i.e., suppression of brain GS activity).

### 3.5. Effect of Chronic VPA Treatment on Brain NH_4_ Concentration and GS Activity

**Figure 3:**
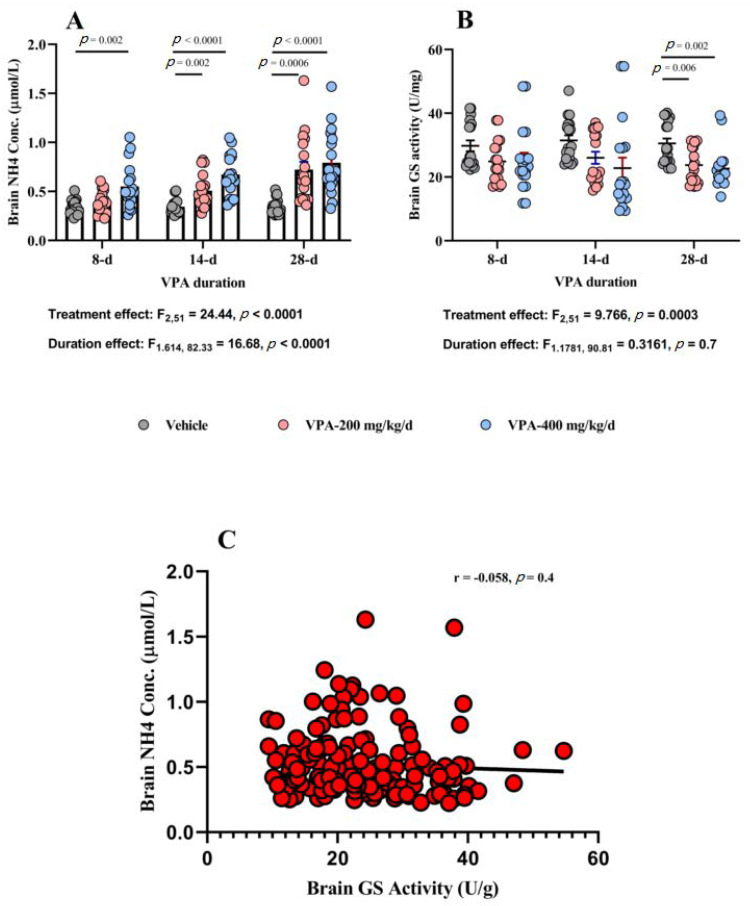
The effect of VPA on brain ammonia (NH_4_) concentration and glutamine synthetase (GS) enzyme activity. (**A**) VPA treatment (F_2,51_ = 24.44, *p* < 0.0001) and duration (F_1.614,82.33_ = 16.68, *p* < 0.0001) both show significant effects on brain NH_4_ concentration by two-way ANOVA (*n* = 6/group). Significant differences in brain NH_4_ concentrations were observed at tested time points between vehicle and both VPA doses. Vehicle vs. VPA 200 mg/kg/d at 14 d (mean difference, 95% CI, *p* value: −0.1630, −0.2698 to −0.05617, *p* = 0.002) and at 28 d (mean difference, 95% CI, *p* value: −0.3726, −0.5811 to −0.1641, *p* = 0.0006). Vehicle vs. VPA 400 mg/kg/d at 8 d (mean difference, 95% CI, *p* value: −0.2159, −0.3543 to −0.07754, *p* = 0.02) and at 14 d (mean difference, 95% CI, *p* value: −0.3281, −0.4579 to −0.1983, *p* < 0.0001) and at 28 d (mean difference, 95% CI, *p* value: −0.4423, −0.6428 to −0.2419, *p* < 0.0001) by Tukey’s multiple comparisons test. (**B**) VPA treatment has significant effect on brain GS activity: F_2,51_ = 9.766, *p* = 0.0003 by two-way ANOVA (*n* = 6/group). Significant differences were observed only at 28 d of treatment between vehicle and VPA 200 mg/kg/d (mean difference, 95% CI, *p* value: 6.803, 1.772 to 11.83, *p* = 0.006) and between vehicle and VPA 400 mg/kg/d (mean difference, 95% CI, *p* value: 7.899, 2.573 to 13.23, *p* = 0.002) by Tukey’s multiple comparisons test. (**C**) No significant correlation was found between brain NH_4_ concentration and brain GS activity: *r* = −0.058, *p* = 0.4.

VPA treatment caused significant treatment and duration effects (*p* < 0.0001 each) on brain NH_4_ concentration and an interaction between the two factors (*p* = 0.004, Table 2 and Figure 3A). VPA at 200 mg/kg/d doses caused significant elevation in brain NH_4_ concentration at 14 d (*p* = 0.002) and at 28 d (*p* = 0.0006) but not in the 8-d group (*p* = 0.1) compared to the vehicle group. Similarly, VPA 400 mg/kg/d was associated with significant increase in brain NH_4_ concentration compared to the vehicle group at 8 d (*p* = 0.002) and at 14 d and 28 d (*p* < 0.0001 each). Individual brain region analysis showed that the elevation in brain NH_4_ concentration is clearly observed in all three areas: PFC, Str, and Cere (*p* < 0.001 each, Supplementary Figures S1A–S3A).

With this elevation in brain NH_4_ concentration, we proceeded by measuring brain GS activity, and, as we expected, brain GS activity was significantly reduced by VPA treatment in a treatment (*p* = 0.0003) but not by duration (*p* = 0.7) effects, and there was no interaction between both factors (*p* = 0.8). Further analysis showed that both VPA 200 and 400 mg/kg/d were associated with robust inhibition of GS activity compared to vehicle group at 28 d of treatment (*p* = 0.006 and *p* = 0.002, respectively, Table 2 and Figure 3B). Neither VPA dose had significant effect on GS activity at 8 or 14 d of treatment. Further analysis of the three brain regions we examined showed that only the PFC showed a significant effect for VPA on GS activity (*p* = 0.0006, Figure S1B) but not Str (*p* = 0.3, Figure S2B) or Cere (*p* = 0.3, Figure S3B).

The elevation in brain NH_4_ concentration and the suppression of brain GS activity were not significantly correlated at the level of the whole brain (r = −0.058, *p* = 0.4, Figure 3C) or in specific regions such as the PFC (*r* = −0.09, *p* = 0.4, Figure S1C) or Str (*r* = −0.26, *p* = 0.056, Figure S2C). Only the Cere showed a slight but significant negative correlation between NH_4_ concentration and GS activity (*r* = −0.286, *p* = 0.03, Figure S3C).

## 4. Discussion

The results of this study showed that chronic VPA treatment was associated with elevation in plasma NH_4_ concentration and behavioral manifestations reminiscent of mild-to-moderate hyperammonemia in animal models of hepatic encephalopathy [31]. This increase in plasma NH_4_ was also accompanied by a concomitant elevation in liver and brain NH_4_ concentration and a suppression of GS enzyme activity.

These findings are in agreement with clinical reports of increased plasma ammonia in the course of VPA treatment as detailed in the introduction. However, despite that over 50% of patients with VPA-hyperammonemia remain asymptomatic [8,10,32,33]. Here, we observed subtle behavioral effects for the increase in plasma NH_4_ levels typical of preclinical reports of dose-dependent reduction in spontaneous locomotor activity in different models of hyperammonemia [34,35]. This apparent contradiction could be related to the ability of animal behavioral assessment scales to capture subtle alterations that would not be easily noticed in routine clinical assessment. A better way to examine the behavioral effects of VPA-associated hyperammonemia could be through testing for specific cognitive domains. Along the same lines, we did not find correlation between the degree of hyperammonemia and Irwin’s total score within hyperammonemia rats only. Our results are in agreement with clinical data where the relationship between behavioral symptoms and NH_4_ concentration has not been established in human cases [6,14].

Next, we examined whether the increase in plasma NH_4_ stems from reduced sequestration into glutamine in liver hepatocytes or brain astrocytes. Our results showed significant negative correlation between the degree of GS inhibition and the elevation in NH_4_ concentration in the liver but not in the brain.

Physiologically, NH_4_ synthesized by gut bacteria, diffuses through the intestinal wall to the capillaries of the portal venules that drain into haptic sinusoids, which end in central venules. These venules group together to form hepatic veins, which drain into the inferior vena cave and systemic circulation [36]. Hepatocytes, alongside haptic sinusoids, are arranged into hexagonal hepatic lobules centered around central venules. Peripherally located hepatocytes are rich in urea cycle enzymes and phosphate-activated glutaminase (PAG) enzyme, while central cells have higher GS enzyme concentrations [37]. Peripheral periportal cells receive NH_4_ first and metabolize it into urea. The urea cycle system has a high capacity for NH_4_ detoxification. However, it also has low affinity, and certain amount of NH_4_ reaches the pericentral hepatocyte GS system. There, NH_4_ is sequestered into glutamine, which is exported to other organs for cellular energy and metabolism [37]. Both the urea cycle and the GS systems ensure low plasma NH_4_ concentration under normal physiological conditions. 

Brain NH_4_, on the other hand, is an integral part of glutamatergic and GABAergic neurotransmission. The brain possesses at least 16 enzymatic pathways for the production of NH_4_, of which three enzymes (PAG, glutamate dehydrogenase, and purine nucleotide cycle) predominate [23]. NH_4_ is generated within glutamatergic and GABAergic neurons through the activity of the PAG enzyme [Gln→Glu + NH_4_]. In GABAergic neurons, PAG-generated glutamate is further converted into GABA by the enzyme glutamic acid decarboxylase. During neuronal firing, equimolar amounts of NH_4_ are generated with glutamate or GABA neurotransmission. The fate of this brain-synthesized NH_4_ is variable. Majority will shuttle back from neurons to astrocytes and form glutamine by GS enzyme [38,39,40,41]. The other portion could diffuse back to the plasma [25], especially if GS capacity is exceeded, since the brain does not have all urea cycle enzymes [42].

As such, the origin of plasma NH_4_ could be traced back to the liver or the brain or to a lesser extent to other organs such as kidney, muscle, or erythrocytes (reviewed in [43]). However, the behavioral aspects of high NH_4_ concentration suggest brain involvement either directly through NH_4_ synthesis or reduced clearance or indirectly through NH_4_ diffusion from plasma to the brain. Since VPA has been shown to increase PAG activity [27], we decided to measure brain GS activity and examine whether it correlates with brain NH_4_ concentration. Here, found that the elevation in brain tissue NH_4_ and suppression of GS activity do not significantly correlate, which suggests that the contribution of GS suppression in the observed increase in brain NH_4_ concentration is modest, and other factors should be examined. Recent data shows GS gene polymorphism (*GLUL* rs10797771) had significant associations with plasma NH_4_ level elevation during VPA-based therapy in a cohort of Japanese pediatric patients with epilepsy [28].

At the regional brain level, we detected a high NH_4_ concentration in all three regions examined. However, concomitant suppression of GS was only evident in the PFC. GS activity in astrocyte culture was reduced by 30% after VPA application [27], and, in agreement with our results, one in-vivo study showed that the effect of VPA on GS activity was region- and time-specific with a robust increase in the hippocampus depending on the duration from prenatal exposure to activity assay [29]. Another study showed no change in hippocampal GS activity after VPA treatment with 200 or 400 mg/kg twice/day for 90 days [44]. Such a simplistic working model entails that VPA directly suppresses GS activity and this causes an increase in brain NH_4_ concentration. Two observations argue against this schema. First, we did not find significant reduction in GS activities in the Str or Cere despite elevation in NH_4_ concentration in both regions. Second, no significant correlation was found between the degree of GS suppression and the elevation in NH_4_ in the PFC, which was the one region that showed GS suppression along with NH_4_ elevation. Taken together, it is likely that other factors besides GS suppression contribute to brain NH_4_ levels. For instance, VPA has been reported to increase the activity of the PAG enzyme [27], which could generate NH_4_ from the breakdown of glutamine. Along the same lines, high plasma NH_4_ could influx across the blood–brain barrier and increase brain NH_4_ concentration. Further studies employing N^15^ spectroscopy [45] to track the source of VPA-induced high brain NH_4_ and to the estimate glutamate–glutamine shuttle enzyme activities are urgently needed.

Regardless of its cause, high brain NH_4_ concentration suppresses the astrocytic glutamate transporter leading to reduced glutamate uptake and increased synaptic glutamate concentration [46,47,48], which seems contradictory to the anticonvulsant effect of VPA. Similarly, the relationship between the behavioral effects of VPA and changes in brain GABA remains unclear [49,50,51]. However, suppression of GS means less glutamine is synthesized by astrocytes and, hence, less glutamine is supplied back to neurons [52]. Given that glutamine is the precursor for glutamate, this reduction in neuronal glutamine could lead eventually to a corresponding reduction in glutamate, and, theoretically, a reduction in overall glutamatergic neuronal excitability, which is a core feature of seizure control. Indeed, VPA treatment in hospitalized bipolar patients was associated with normalization of high brain glutamate (glutamate + glutamine GLX) as measured by MR spectroscopy [53]. Moreover, the effect of VPA on the glutamate transporter is also region specific. One study found no effect of chronic VPA treatment on glutamate transport protein expression in the frontal or parietal cortices or in the cerebellum but found a significant increase in the hippocampus [44]. Whether VPA suppression of brain GS activity plays a role in its anticonvulsant mechanism of action remains to be investigated.

The results of this study should be viewed in light of its limitations. First, we used two different VPA doses to ensure adequate VPA blood levels close to those reported in clinical practice. We measured peak and trough VPA concentrations in a subgroup of animals. VPA levels in our report (peak at 20 min post dose: 250 ± 28.87 µg/mL and trough at 12 h post dose: 6.21 ± 1.63 µg/mL) are consistent with pharmacokinetic studies showing that the time required for the maximum concentration of VPA 150 mg/kg was 41 min followed by a rapid decline [54]. One previous study reported that the VPA level at 120 min after a single administration of 360 mg/kg to female Wistar rats was 78.7 ± 29.4 µg/mL [55]. Therapeutic VPA concentrations in human studies show wide variability and range between 50 and 100 µg/mL [56]. However, the correlation between VPA dose and plasma level is poor [57,58]. The level of VPA at 60 min post last dose was 133 ± 36 µM/mL in male Wistar rats that were administered VPA 200 mg/kg twice/d for 90 days [44]. Second, we measured the VPA concentration in a subgroup of animals, hence, we could not examine correlation between plasma VPA and NH_4_ concentration. However, most [11,12,14], but not all [8,9,32,59], clinical studies reported that VPA and NH_4_ concentrations are not necessarily corelated. Third, we only quantified GS activity as the primary NH_4_ metabolizing enzyme in the brain. Including other enzymes such as PAG and alpha ketoglutarate dehydrogenase and measuring cerebrospinal fluid (CSF) NH_4_ could explain the discrepancy between NH_4_ elevation and GS suppression. Finally, we only measured NH_4_ concentration and GS enzyme activity in Str, Cere, and PFC. We selected these specific regions because of their critical involvement in locomotor activity, motor coordination, and cognitive functions. All of which are impaired in symptomatic hyperammonemia [16,34,35]. However, other relevant areas such as the hippocampus and amygdala are also intimately related to seizure and mood disorders and should be investigated in future studies. Despite these limitations, our results bring new insights into the mechanism of VPA-induced hyperammonemia through highlighting the effect of VPA on brain and liver GS and the potential contribution of the brain to the phenomena of VPA-induced high plasma NH_4_ levels and call for further investigations into the contribution of brain NH_4_ into the observed elevation in plasma NH_4_ concentration.

## 5. Conclusions

The current study highlights two interesting findings. First, hyperammonemia in the course of chronic VPA treatment is not limited to the plasma. Indeed, brain NH_4_ concentration also increases and may contribute, at least in part, to the clinical picture. Second, the potential mechanistic role of GS suppression in VPA-induced hyperammonemia does not fully explain the elevation of brain NH_4_. This observation does not come as a surprise, given that brain NH_4_ regulation is influenced by other enzymes and several other factors such as NH_4_ transporters, acid-base balance, and exchange of glutamine and branched chain amino acids across blood brain barrier. Future studies should explore all these aspects of brain ammonia economy under VPA treatment.

## Figures and Tables

**Table 1:** The effect of valproic acid (VPA) on Irwin’s behavioral measures. Data presented at mean ± SEM (*n* = 6–7/group).

Behavioral Measures	Treatment	8 d (Mean ± SEM)	14 d (Mean ± SEM)	28 d (Mean ± SEM)
Total Score	Vehicle	22.6 ± 1.9	23.0 ± 1.1	19.7 ± 2.8
	VPA 200 mg/kg/day	16.6 ± 1.5	17.5 ± 1.1	17.7 ± 1.6
	VPA 400 mg/kg/day	15.6 ± 1.2	14.3 ± 1.0	12.3 ± 0.3
Corneal Reflex	Vehicle	6.286 ± 0.68	5.66 ± 0.61	6.0 ± 0.51
	VPA 200 mg/kg/day	5.0 ± 0.44	6.25 ± 0.45	4.75 ± 0.36
	VPA 400 mg/kg/day	4.66 ± 0.42	4.0 ± 0.0	4.0 ± 0.0
Pinna Reflex	Vehicle	5.0 ± 1.12	5.66 ± 0.61	4.33 ± 0.33
	VPA 200 mg/kg/day	1.33 ± 0.66	1.5 ± 0.32	1.5 ± 0.82
	VPA 400 mg/kg/day	1.0 ± 0.44	0.33 ± 0.33	0.0 ± 0.0
Positional Passivity	Vehicle	4 ± 0.89	4.66 ± 0.84	6.0 ± 0.73
	VPA 200 mg/kg/day	6.33 ± 0.33	5.5 ± 0.32	7.75 ± 0.25
	VPA 400 mg/kg/day	8.0 ± 0.0	7.33 ± 0.42	8.0 ± 0.0
Pain Reflex	Vehicle	7.33 ± 0.66	7.0 ± 0.44	6.66 ± 0.42
	VPA 200 mg/kg/day	4.0 ± 0.73	4.25 ± 0.79	3.75 ± 1.09
	VPA 400 mg/kg/day	2.0 ± 0.51	2.66 ± 1.22	0.33 ± 0.33

**Table 2:** The effect of VPA on plasma, liver and brain ammonia (NH_4_) concentration, and on liver and brain, prefrontal cortex (PFC), striatal (Str), and cerebellar (Cere) NH_4_ concentration and glutamine synthetase (GS) enzyme activity. Data presented as mean ± SEM (*n* = 6/group).

Behavioral Measures	Treatment	8 d (Mean ± SEM)	14 d (Mean ± SEM)	28 d (Mean ± SEM)
Plasma NH4 Conc (μmol/L)	Vehicle	67.98 ± 8.9	72.5 ± 6.4	68.5 ± 8.1
	VPA 200 mg /kg/day	159.3 ± 14.6	246.4 ± 34.6	182.1 ± 15.6
	VPA 400 mg/kg/day	215.8 ± 22.7	176.2 ± 16.9	182.3 ± 21.0
Liver NH4 Conc (μmol/mg)	Vehicle	0.34 ± 0.01	0.33 ± 0.01	0.34 ± 0.01
	VPA 200 mg /kg/day	0.35 ± 0.03	0.28 ± 0.04	0.34 ± 0.02
	VPA 400 mg/kg/day	0.38 ± 0.03	0.38 ± 0.01	0.6 ± 0.09
Liver GS activity (U/mg)	Vehicle	56.9 ± 5.2	54.4 ± 5.0	55.2 ± 4.0
	VPA 200 mg /kg/day	32.4 ± 0.8	52.8 ± 1.5	58.2 ± 3.8
	VPA 400 mg/kg/day	44.2 ± 2.1	39.6 ± 0.8	31.7 ± 5.7
Brain NH4 Conc (μmol/mg)	Vehicle	0.33 ± 0.01	0.34 ± 0.01	0.34 ± 0.01
	VPA 200 mg /kg/day	0.39 ± 0.02	0.50 ± 0.03	0.72 ± 0.08
	VPA 400 mg/kg/day	0.55 ± 0.05	0.67 ± 0.04	0.79 ± 0.07
Brain GS activity (U/mg)	Vehicle	29.7 ± 1.7	31.4 ± 1.5	30.5 ± 1.5
	VPA 200 mg /kg/day	24.8 ± 1.5	26.0 ± 1.8	23.71.3
	VPA 400 mg/kg/day	25.1 ± 2.4	22.7 ± 2.4	22.6 ± 1.5

## Data Availability

Data files are available upon request.

## Supplementary Materials

The following are available online at https://www.mdpi.com/2076-3425/10/10/759/s1, Figure S1: Prefrontal Cortex ammonia concentration and glutamine synthetase enzyme activity. Figure S2: Striatal ammonia concentration and glutamine synthetase enzyme activity. Figure S3: Cerebellar ammonia concentration and glutamine synthetase enzyme activity.
