# Supplementary material for: Chronic Valproic Acid Administration Increases Plasma, Liver, and Brain Ammonia Concentration and Suppresses Glutamine Synthetase Activity
Source: Brain Sci. 2020 Oct 21;10(10):759. doi: 10.3390/brainsci10100759 (PMC7589689; doi:10.3390/brainsci10100759)
Supplement: Supplementary file 1 [file brainsci-10-00759-s001.pdf]

Chronic Valproic Acid Administration Increases Plasma, Liver, and Brain Ammonia Concentration and Suppresses Glutamine Synthetase Activity

Abdelnasr A. Badawy, Rasha Elghaba, Mohamed Soliman, Abdelaziz M. Hussein, Sana A. AlSadrah, Amira Awadalla and Osama A. Abulseoud

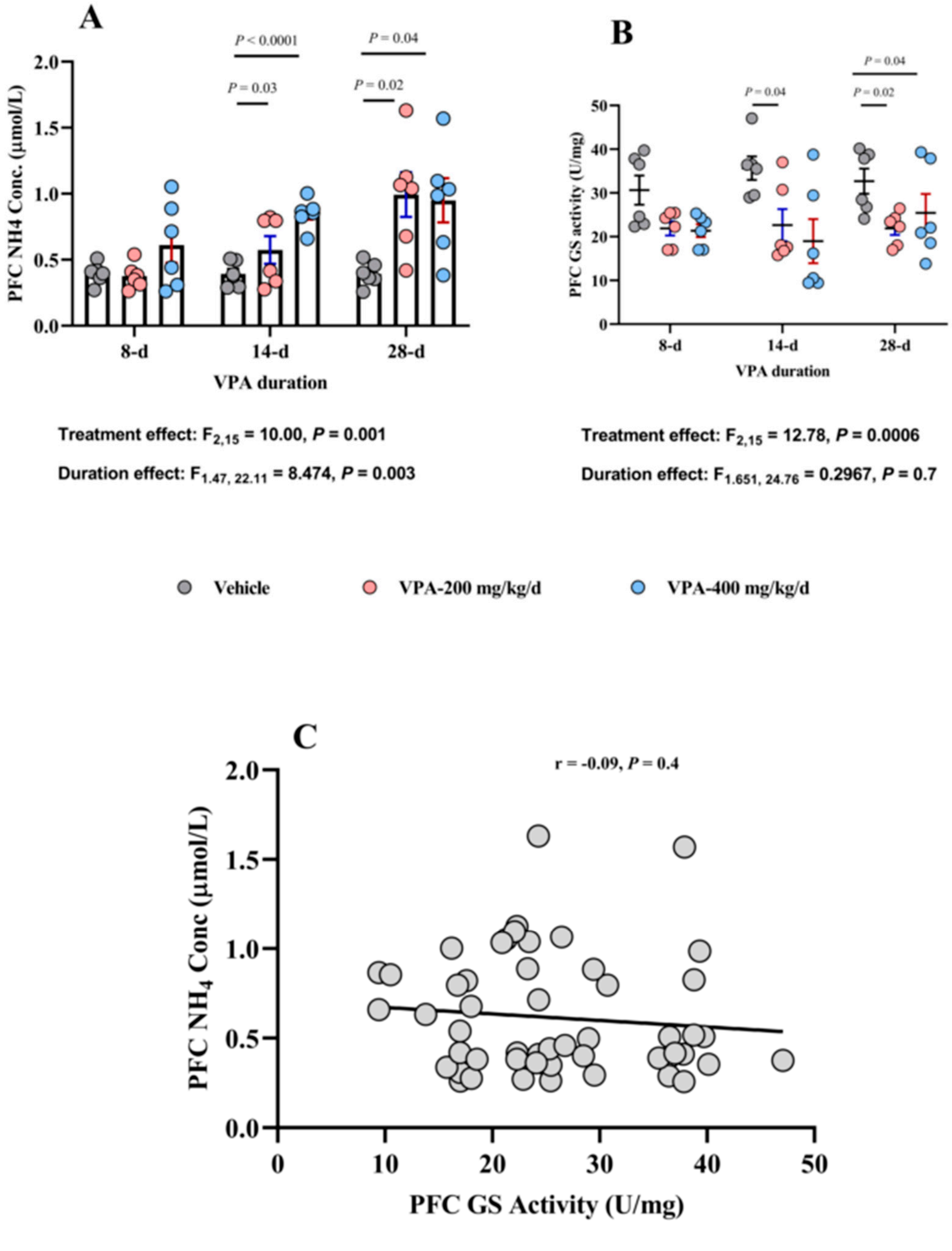

Figure S1: The effect of VPA on prefrontal cortex (PFC) ammonia (NH_4) concentration and glutamine synthetase (GS) enzyme activity. (**A**) VPA treatment ($F_{2,45}$ = 10.0, P = 0.001) and duration ($F_{1.474,22.11}$ = 8.474, P = 0.003) both has significant effects on PFC NH_4 concentration by two-way ANOVA (*n* =6/group). Significant differences in PFC NH_4 concentrations were observed at tested time points between vehicle and both VPA doses. Vehicle vs VPA 400mg/kg/d at 14-d (mean difference, 95% CI,

P value: -0.4563, -0.6204 to -0.2923, P<0.0001) and at 28-d between vehicle and VPA 200mg/kg/d (mean difference, 95% CI, P value: −0.6008, −1.148 to −0.05369, P=0.03) and between Vehicle vs VPA 400mg/kg/d (mean difference, 95% CI, P value: −0.5575, −1.097 to −0.01839, P = 0.044) by Tukey's multiple comparisons test. (**B**) VPA treatment has significant effect on PFC GS activity: $F_{2,15}$ = 12.78, P = 0.0006 by two-way ANOVA (*n* = 6/group). Significant differences were observed between vehicle and VPA 200mg/kg/d at 14-d treatment (mean difference, 95% CI, P value: 13.01, 0.4107 to 25.61, P= 0.043) and at 28-d treatment points (mean difference, 95% CI, P value: 10.78, 1.419 to 20.13, P = 0.02) and between vehicle and VPA 400mg/kg/d at 14-d treatment (mean difference, 95% CI, P value: 16.70, 0.2053 to 33.18, P = 0.047) by Tukey's multiple comparisons test. (**C**) No significant correlation between PFC NH_4 concentration and brain GS activity: r =-0.09, P = 0.4.

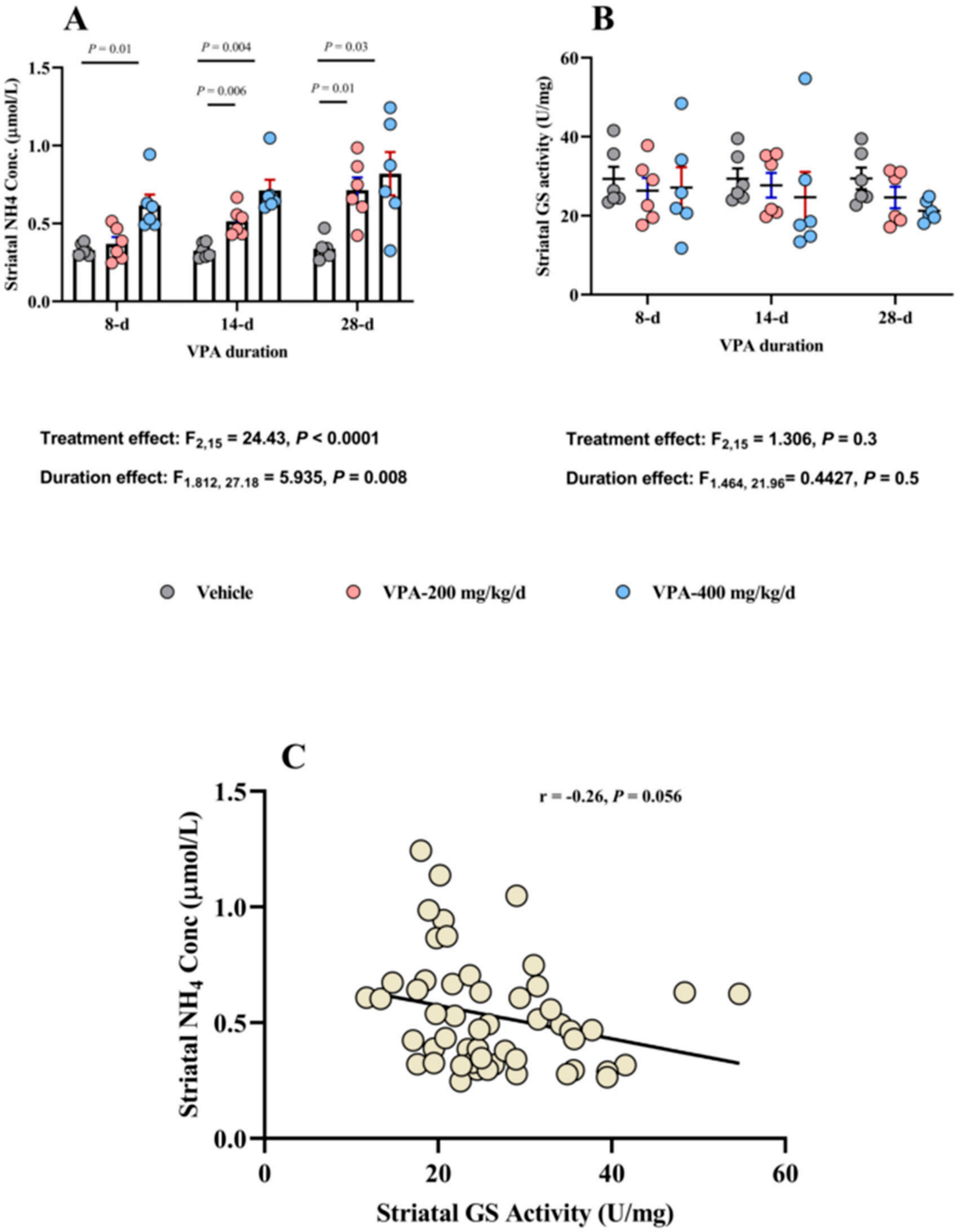

Figure S2: The effect of VPA on striatal (Str) ammonia (NH_4) concentration and glutamine synthetase (GS) enzyme activity. (**A**) VPA treatment ($F_{2,15}$ = 24.43, P < 0.0001) and duration ($F_{1.812,27.18}$ = 5.935, P =

0.008) both have significant effects on Str NH_4 concentration by two-way ANOVA (n = 6/group). Significant differences in PFC NH_4 concentrations were observed at tested time points between vehicle and both VPA doses. Vehicle vs VPA 200mg/kg/d at 14-d (mean difference, 95% CI, P value: −0.1891, −0.3113 to −0.06684, P = 0.006) and at 28-d (mean difference, 95% CI, P value: −0.3759, −0.6369 to −0.1150, P = 0.01). Vehicle vs VPA 400mg/kg/d at 8-d (mean difference, 95% CI, P value: −0.2865, −0.5110 to −0.06191, P = 0.01) and at 14-d (mean difference, 95% CI, P value: −0.3862, −0.6059 to −0.1664, P = 0.004) and at 28-d (mean difference, 95% CI, P value: −0.4805, −0.9279 to −0.03299, P = 0.03) by Tukey's multiple comparisons test. (**B**) Neither VPA treatment nor duration has significant effect on Str GS activity by two-way ANOVA (n = 6/group). (**C**) Non-significant trend for negative correlation between Str NH_4 concentration and brain GS activity: r = −0.26, P = 0.056

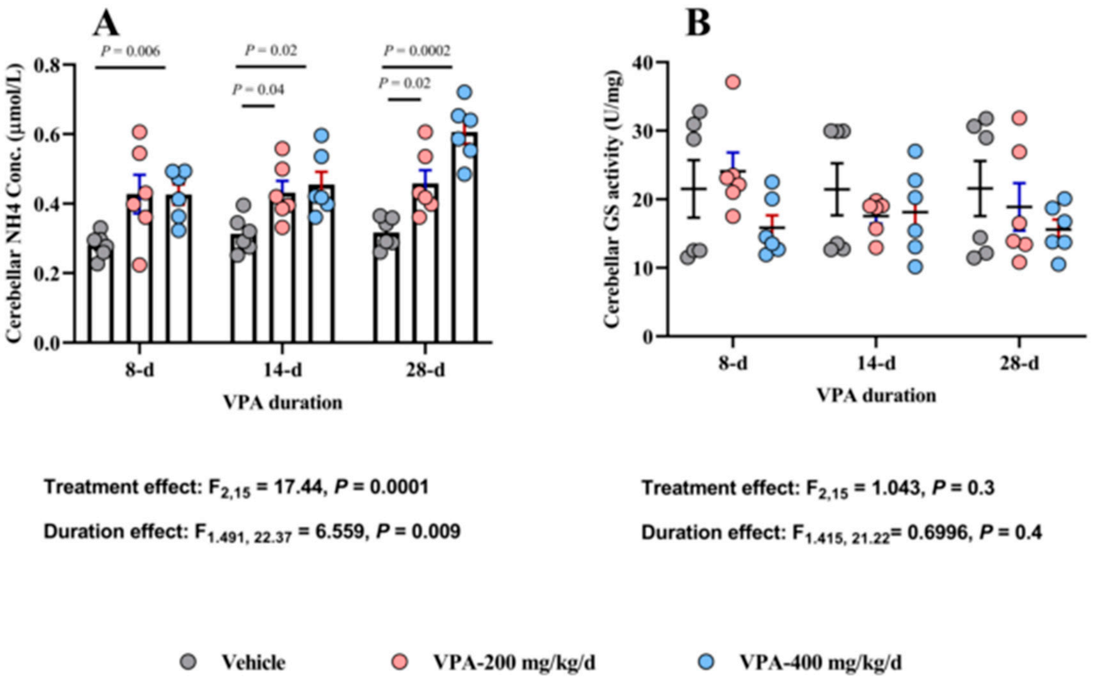

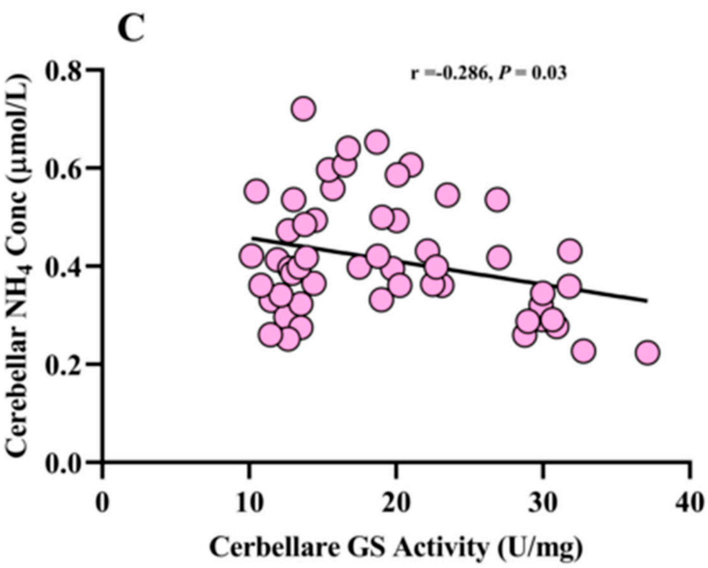

Figure S3: The effect of VPA on cerebellar (Cere) ammonia (NH_4) concentration and glutamine synthetase (GS) enzyme activity. (**A**)VPA treatment ($F_{2,15}$ = 17.44, P=0.0001) and duration ($F_{1.491,22.37}$ =

4.559, P = 0.009) both have significant effects on Cere NH_4 concentration by two-way ANOVA (n = 6/group). Significant differences in Cere NH_4 concentrations were observed at tested time points between vehicle and both VPA doses. Vehicle vs VPA 200mg/kg/d at 14-d (mean difference, 95% CI, P value: −0.1186, −0.2314 to −0.005808, P=0.04) and at 28-d (mean difference, 95% CI, P value: −0.1410, −0.2640 to −0.01797, P = 0.02) and between Vehicle vs VPA 400mg/kg/d at 8-d (mean difference, 95% CI, P value: −0.1453) and at 28-d (mean difference, 95% CI, P value: −0.2891, −0.3998 to −0.1784, P = 0.0002) by Tukey's multiple comparisons test. (**B**) Neither VPA treatment nor duration has significant effect on Cere GS activity by two-way ANOVA (n = 6/group). (**C**) Significant negative correlation between Cere NH_4 concentration and brain GS activity: r = −0.286, P = 0.03.
